# A potent and broad CD4 binding site neutralizing antibody with strong ADCC activity from a Chinese HIV-1 elite neutralizer

**DOI:** 10.1038/s41421-025-00808-x

**Published:** 2025-06-10

**Authors:** Yingdan Wang, Ping Ji, Qianying Liu, Nannan Jia, Yunping Ma, Tianyi Yuan, Palizhati Rehati, Jiali Chen, Yumei Wen, Fan Wu, Jinghe Huang

**Affiliations:** 1 Key Laboratory of Medical Molecular Virology (MOE/NHC/CAMS) and Shanghai Institute of Infectious Disease and Biosecurity, Shanghai Frontiers Science Center of Pathogenic Microorganisms and Infection, School of Basic Medical Sciences, Fudan University, Shanghai, China; 2 Shanghai Immune Therapy Institute, Shanghai Jiao Tong University School of Medicine Affiliated Renji Hospital, Shanghai, China; 3 School of Basic Medical Sciences, Southwest Medical University, Luzhou, Sichuan China; 4 Xuchang Center for Disease Control and Prevention, Henan, China

**Keywords:** Immunology, Molecular biology, Molecular modelling

## Abstract

The discovery of broadly neutralizing antibodies (bNAbs) that target conserved epitopes on the HIV-1 envelope glycoprotein (Env) has garnered significant attention for its potential in the development of effective therapeutic and vaccine strategies. In this study, we isolated and characterized a CD4 binding site (CD4bs) antibody, FD22, from an elite neutralizer in China who had been infected with a clade B virus through contaminated blood plasma for 23 years. The heavy chain of FD22 was derived from a rarely reported IGHV3-30 germline gene and exhibited an exceptionally high degree of somatic hypermutation (SHM) (37%), along with a long and unique CDRH3 loop of 20-amino acids. FD22 exhibited potent and broad neutralizing activity, comparable to that of the well-known bNAb VRC01. It effectively neutralized 82% of a panel of 145 diverse HIV-1 pseudoviruses, including the two major circulating strains in China, CRF01_AE and CRF07_BC. FD22 bound strongly to HIV-1-infected cell lines, efficiently engaged FcγRIIIa receptors, triggered NK cell degranulation and the release of key cytokines such as IFN-γ and β-chemokines, and robustly induced antibody-dependent cellular cytotoxicity (ADCC) against HIV-1-infected target cells. Structural prediction for FD22 and the HIV Env SOSIP trimer performed by AlphaFold3, site-mutagenesis, and autologous virus reverse mutation assays revealed that the epitope of FD22 spans key CD4 binding site, including Loop D, the CD4 binding loop (CD4 BLP), and the V5 Loop. The unique long CDRH3 loop of FD22 interacts with the CD4 binding site through its negatively charged residue R102, distinguishing it from other CD4bs antibodies. Our findings provide valuable insights into the mechanisms of FD22 in viral neutralization and ADCC. The dual functionality of FD22 enhances its potential as a promising therapeutic antibody and offers new avenues for designing CD4bs-targeting vaccines with enhanced ADCC capabilities.

## Introduction

Broadly neutralizing antibodies (bNAbs) for HIV-1 arise in rare individuals known as elite neutralizers and target specific regions of the viral envelope glycoprotein (Env), including the CD4 binding site (CD4bs), glycan-associated loops, and the gp120‒gp41 interface^1,2^. These antibodies are capable of neutralizing a wide range of HIV strains and have demonstrated potential in protecting nonhuman primates from infection and controlling HIV in humans after treatment interruption^3–6^. Additionally, bNAbs can eliminate infected cells through Fc-mediated mechanisms and enhance immune responses by forming immune complexes^7–10^. Their distinctive features position bNAbs as promising tools for HIV-1 prevention and therapy and potentially for achieving a functional cure, as evidenced by natural HIV controllers^11,12^.

The CD4bs is the primary site of viral attachment to host CD4^+^ T cells, making it a vulnerable target for antibody-mediated neutralization. Antibodies directed against this site can block viral entry across diverse HIV-1 strains, highlighting their therapeutic and preventive potential^13–15^. Efforts to elicit CD4bs-targeting bNAbs through vaccination have led to promising strategies. Notably, a polyvalent DNA prime-protein boost vaccine based on gp120 has shown potential for eliciting CD4bs antibodies capable of neutralizing tier 2 HIV strains^16^. Germline-targeting approaches have further informed immunogen design by identifying antibody gene lineages predisposed to develop into CD4bs bNAbs^17,18^. The IGHV1-2*02 gene segment has been repeatedly identified as the heavy chain precursor of potent CD4bs-directed bNAbs, such as VRC01^19^ and 3BNC117^20^. These findings have guided the development of immunogens specifically designed to engage and mature these germline B-cell receptors. However, the potential of alternative germline lineages to give rise to CD4bs-targeting bNAbs remains largely unexplored, representing a critical gap in our understanding of antibody diversity and bNAb development.

In this study, FD22, a CD4bs antibody isolated from an HIV-1 elite neutralizer in China, was identified and characterized. Derived from the IGHV3-30 germline with high somatic hypermutation (37%) and a 20-amino-acid CDRH3 loop, FD22 exhibits potent and broad neutralizing activity, neutralizing 82% of a 145-strain HIV-1 panel, comparable to the neutralization activity of VRC01. It also mediates robust antibody-dependent cellular cytotoxicity (ADCC) against HIV-1-infected cells. Structural analyses revealed interactions at key Env loops, such as Loop D, the CD4 binding loop (CD4 BLP), and the V5 Loop, along with the unusually long CDRH3 loop of FD22, particularly the positively charged residue R102, offering insights into its neutralization and ADCC mechanism. This highlights the potential of FD22 for advancing HIV-1 therapy and vaccine development using a new germline-targeted strategy.

## Results

### Isolation and characterization of FD22

**Fig. 1:**
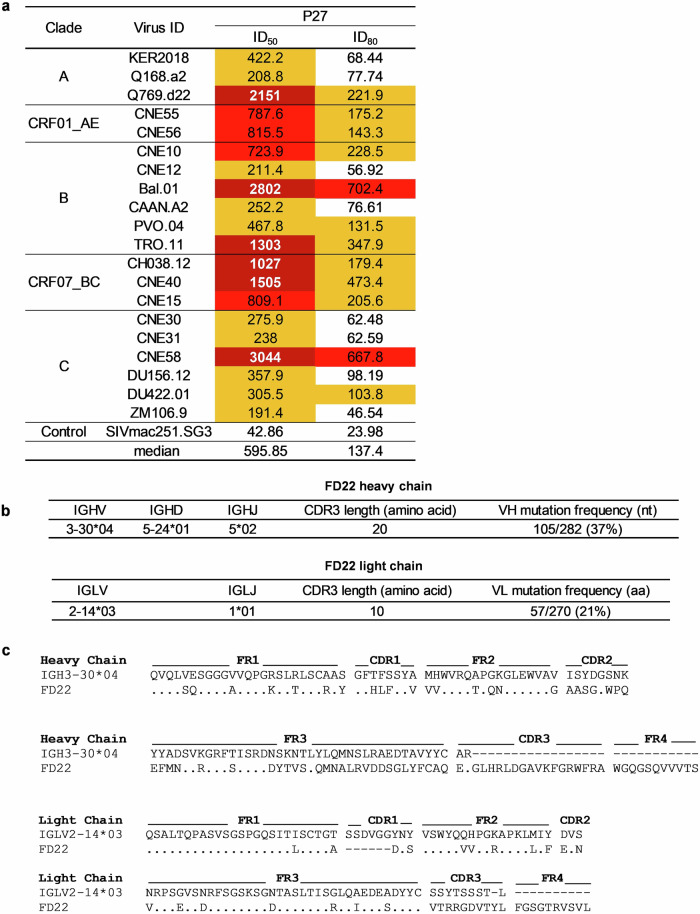
Isolation and characterization of the antibody FD22 from patient P27. **a** Neutralization titers (ID_50_, 50% inhibitory dilution and ID_80_, 80% inhibitory dilution) of the serum from patient P27 against 20 pseudoviruses representing clades A, CRF01_AE, B, CRF07_BC, and C. **b** Germline gene origins of the heavy- and light-chain variable regions of FD22 identified using the IMGT database (http://imgt.org). **c** Sequence alignment of the heavy- and light-chain variable regions of FD22.

We identified an elite neutralizer patient, P27, who has been diagnosed with HIV-1 for 23 years and carries a clade B virus. Notably, P27 did not receive antiretroviral therapy (ART) until the time of blood sampling. At the time of lymphocyte apheresis, P27 had a viral load of 6660 copies/mL and a CD4^+^ T-cell count of 559 cells/μL. The serum from P27 exhibited broad and potent neutralizing activity, achieving 100% coverage and a median 50% inhibitory dilution (ID_50_) of 595.85 (Fig. 1a).

To identify the monoclonal antibodies responsible for the serological neutralizing activity of P27, IgG^+^ memory B cells were isolated from peripheral blood mononuclear cells (PBMCs) and cultured in 384-well plates, as previously described^21^. In a microneutralization assay, we identified an antibody, FD22, which exhibited potent anti-HIV-1 neutralizing activity against two strains, Bal.26 and MN.3. The FD22 heavy chain originated from the rarely reported IGHV3-30 germline gene and exhibited a remarkably high somatic hypermutation (37%), featuring a distinct 20-amino-acid CDRH3 loop (Fig. 1b, c). The light chain derived from the IGLV2-14*03 germline gene and had a mutation frequency of 21% (Fig. 1b, c).

**Fig. 2:**
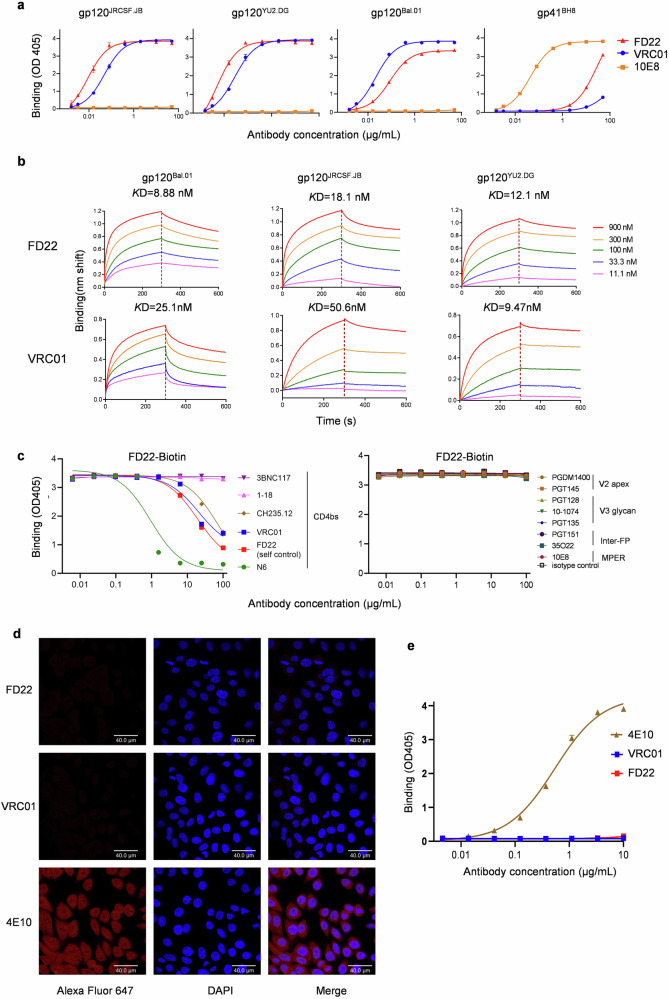
Binding activity of FD22. **a** The binding of FD22 to the HIV-1 gp120 and gp41 proteins was measured by ELISA, with VRC01 and 10E8 serving as controls. The results are presented as the means ± SD, with error bars representing variability. **b** The binding affinity of FD22 for selected HIV-1 gp120 proteins was analyzed using BLI, with VRC01 included for comparison. The *K*D values for binding affinity are displayed above each graph. **c** Competitive binding analysis was performed to assess the binding of FD22-biotin to gp120^JRCSF.JB^ in the presence of antibodies targeting CD4bs, V2 apex, V3-glycan, inter-FP, or MPER. **d** The HEp-2 assay was used to evaluate the potential autoreactivity of FD22. Representative results from two independent experiments are shown. Scale bars, 40 μm. **e** ELISA was conducted to examine the binding of FD22 to the self-antigen cardiolipin. Optical density (OD) values at 405 nm are displayed in duplicate.

FD22 binds robustly to the HIV-1 gp120 proteins from the JRCSF.JB, YU2.DG and BaL.01 strains, with binding levels comparable to those of the CD4bs bNAb VRC01 (Fig. 2a). Concentration‒response curves further revealed that FD22 binds HIV-1 gp120 proteins with higher binding affinity and lower affinity constant (*K*D) values than VRC01 does (Fig. 2b; Supplementary Fig. S1). Competition ELISA experiment revealed that FD22 competes with CD4bs antibodies for binding to the gp120 protein, with the binding strength ranked from strongest to weakest as follows: N6 > VRC01 > CH235.12 (Fig. 2c). However, they did not compete with antibodies targeting other regions, including the V2 apex (PGDM1400, PGT145), V3-glycan supersite (PGT128, 10-1074, PGT135), gp120-gp41 interface (PGT151, 35O22), or membrane-proximal external region (MPER) (10E8) (Fig. 2c). Interestingly, the CD4bs antibodies 1-18 and 3BNC117 did not affect the binding of FD22 to gp120, suggesting that FD22 binds to gp120 in a manner distinct from both 1-18 and 3BNC117.

The frequency of autoreactive antibodies is high during the development of HIV bNAbs^22^. For example, MPER-targeting bNAbs such as 2F5 and 4E10 have been shown to exhibit polyreactivity and autoreactivity^23^. To assess the self-reactive potential of FD22, we evaluated its binding to the HEp-2 cell line and cardiolipin. The results showed that FD22 did not bind to HEp-2 epithelial cells (Fig. 2d) or cardiolipin (Fig. 2e), suggesting that FD22 does not exhibit autoreactivity or polyreactivity — properties commonly observed in some HIV-specific antibodies that could limit their therapeutic or prophylactic potential. This lack of reactivity with self-antigens further supports the potential of FD22 as a safe and targeted therapeutic candidate.

### Neutralizing activity and suppression infection of reactivated latent HIV by FD22

**Fig. 3:**
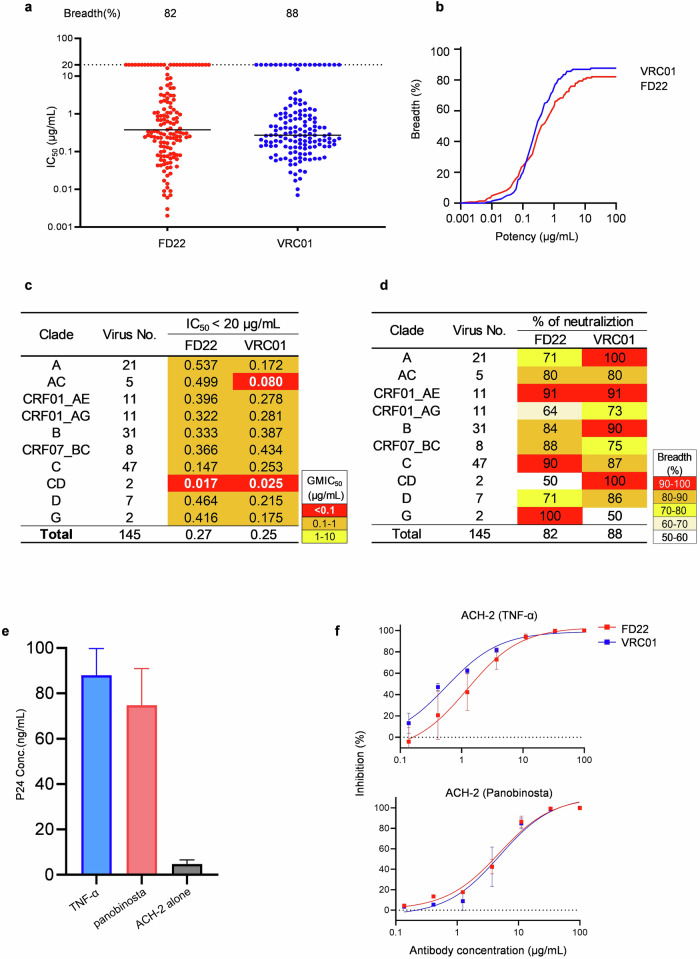
Neutralizing activity and suppression of infection of reactivated latent HIV by FD22. **a**, **b** Neutralization potency (**a**) and breadth (**b**) of FD22, in comparison with VRC01, against a 145-isolate Env-pseudovirus panel. **c**, **d** The neutralization activity (**c**) and percentage (**d**) of FD22 were evaluated against different clades of HIV-1 pseudoviruses from a 145-virus panel, including the two predominant circulating strains in China, CRF01_AE and CRF07_BC. VRC01 served as a control. **e** Activation of HIV-1 latency in ACH-2 cells. HIV-1 latency reversal in ACH-2 cells was induced by treatment with TNF-α or panobinostat for 48 h. Viral reactivation was assessed by quantifying p24 antigen concentrations using a standardized ELISA. **f** Inhibitory activity of FD22 against latent HIV-1 in ACH-2 cells activated by TNF-α or panobinostat, as measured in TZM-bl cells. ACH-2 cells were treated with TNF-α (10 ng/mL) or panobinostat (1 μM) to induce HIV-1 reactivation. After activation, FD22 was added at various concentrations to assess its inhibitory activity. Reactivated virus production was measured by coculturing supernatants from treated ACH-2 cells with TZM-bl indicator cells.

Next, we evaluated the neutralization breadth and potency of FD22 using a panel of 145 pseudotyped viruses. FD22 exhibited broad neutralization, effectively neutralizing 82% of the pseudoviruses with a geometric mean (GM) IC_50_ of 0.27 µg/mL, whereas VRC01 neutralized 88% of the panel with a GM IC_50_ of 0.25 µg/mL (Fig. 3a–c; Supplementary Table S1). Additionally, the IC_50_ values of FD22 against diverse isolates showed a strong correlation with those of VRC01 (Supplementary Fig. S2). CRF01_AE and CRF07_BC are the two major circulating HIV-1 strains in China. CRF01_AE has been associated with lower CD4^+^ T-cell counts and poorer immune recovery in Chinese patients^24^, while CRF07_BC exhibits enhanced transmission capability compared to HIV-1 subtypes B and C^25^. Notably, CRF07_BC contains a more heavily glycosylated V1 region^26^, which is thought to hinder the binding of many bNAbs. Given the unique biological characteristics of CRF01_AE and CRF07_BC, as well as their increasing epidemiological prevalence in China, evaluating the neutralization efficacy of FD22 against these strains is particularly significant. Although FD22 was isolated from a Chinese patient P27 infected with the B strain, it exhibited an exceptional neutralization breadth against eleven CRF01_AE strains and eight CRF07_BC strains, with neutralization rates of 91% and 88%, respectively (Fig. 3d). In comparison, VRC01 exhibited similar neutralization breadth against CRF01_AE (91%) but neutralized only 75% of CRF07_BC strains (Fig. 3d). These results highlight FD22’s broad neutralization profile, particularly against the more resistant CRF07_BC lineage, underscoring its potential for use in regions where this subtype is prevalent.

To evaluate the potential of the FD22 antibody to inhibit infection of reactivated latent HIV-1, we used the HIV-1 latent cell line ACH-2 as a model system, with reactivation induced by TNF-α and panobinostat^27^. Extracellular p24 was significantly increased in the supernatants of ACH-2 in response to treatment with TNF-α and panobinosta (from a basal level of 4.69 ng/mL to 87.93 ng/mL and 74.81 ng/mL, respectively) (Fig. 3e), indicating efficient induction of latent HIV-1. FD22 exhibited potent inhibitory activity against the infection of target cells by HIV-1 reactivated from latent reservoirs, with efficacy comparable to the potent HIV antibody VRC01 (Fig. 3f), suggesting its potential to restrict new rebounds of infection following latency reversal. Taken together, these data suggest that FD22 is a broad and potent CD4bs antibody identified from a Chinese HIV-1-infected donor. It demonstrates neutralization activity and inhibition of infection by reactivated latent HIV, comparable to that of VRC01. Given the marked neutralization breadth and potency of FD22 against the predominant circulating strains CRF01_AE and CRF07_BC in China, it holds promise as an effective therapeutic candidate for treating HIV-1 infections in the region.

### Induction of FcγRIIIa stimulation by FD22

**Fig. 4:**
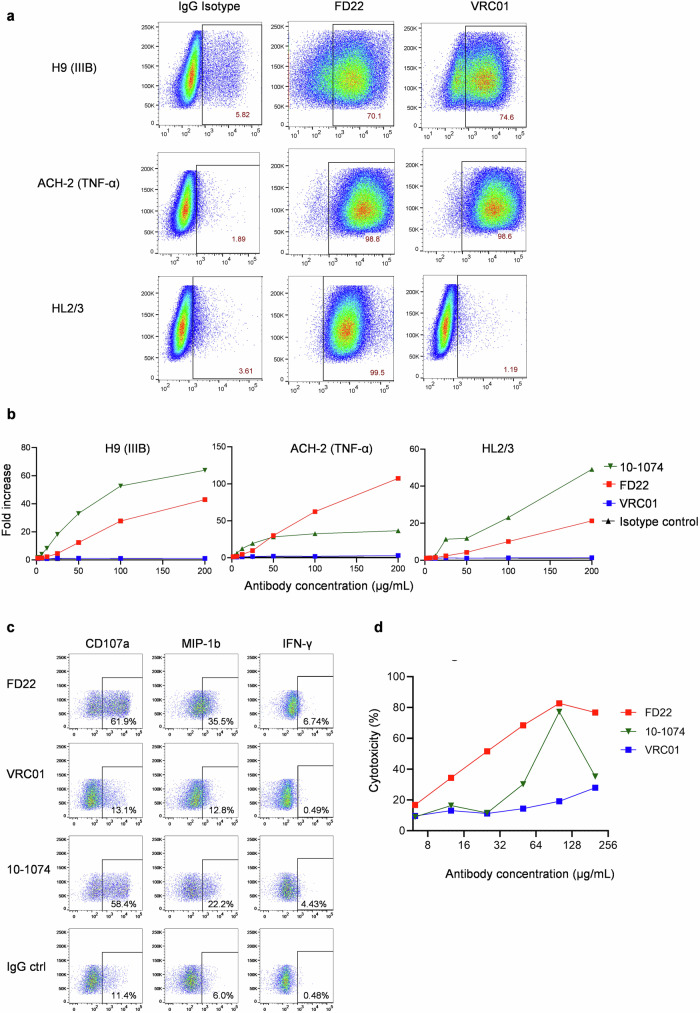
Induction of FcγRIII stimulation, NK activation, and ADCC by FD22. **a** Flow cytometry analysis of FD22 binding to the cell surface of H9 (IIIB) cells, TNF-α reactivated ACH-2 cells, and HL2/3 cells. VRC01 and the IgG isotype were used as controls. **b** FD22-mediated ADCC was evaluated using Jurkat-Lucia NFAT-CD16a reporter cells in the presence of H9 (IIIB) cells, TNF-α reactivated ACH-2 cells, or HL2/3 cells. FcγRIIIa signaling activity was quantified by measuring the luciferase signal from an NFAT-dependent reporter, with isotype control-induced signaling set to 1 for comparison. The ADCC V3-specific antibody 10-1074 served as a positive control. **c** Degranulation of NK cells upon FD22 engagement, as measured by CD107a surface expression. Intracellular staining for MIP-1β and IFN-γ release from NK cells in response to FD22. The data are representative of at least three independent experiments in which NK cells were isolated from the peripheral blood of healthy donors. **d** ADCC activity of FD22 against HIV-1-infected dells. The ADCC activity of FD22 was assessed using the LDH release cytotoxicity assay. H9 (IIIB) cells served as target cells, while PBMCs from healthy donors were used as effector cells at an E:T ratio of 6:1. FD22 was tested at concentrations ranging from 6.25 to 200 μg/mL.

The binding of FD22 to the HIV-1(IIIB)-infected H9 cell line, TNF-α-activated ACH-2 latent HIV-1 cells, and HL2/3 cells expressing HIV Env was evaluated using flow cytometry. FD22 exhibited strong binding to all three cell lines (Fig. 4a). In contrast, VRC01 showed strong binding to H9 (IIIB) and ACH-2 cells but weak binding to HL2/3 cells. The difference in binding between FD22 and VRC01 to HL2/3 cells suggests that FD22 may target a slightly distinct or more accessible epitope within the CD4 binding site.

When incubated with these three cell lines and co-cultured with Jurkat-Lucia NFAT-CD16a (FcγRIIIa) cells, which express an NFAT-luciferase reporter activated by FcγRIIIa stimulation^28^, FD22 induced a robust FcγRIIIa response comparable to that of the potent ADCC inducer 10-1074 (Fig. 4b). This indicates that FD22 effectively engages FcγRIIIa-expressing immune cells, a key mechanism for ADCC-mediated viral clearance. In contrast, VRC01 failed to trigger FcγRIIIa stimulation, highlighting a notable difference in effector function between the FD22 and VRC01.

### Activation of NK cells and ADCC of FD22

To further investigate the ADCC potential of FD22, natural killer (NK) cells were isolated from the PBMCs of healthy donors and used as effector cells. Antibody-dependent NK cell degranulation was assessed by measuring CD107a expression on the surface of NK cells following incubation with H9 (IIIB) target cells and antibodies. The results demonstrated that FD22 induced robust NK cell degranulation in the presence of H9 (IIIB) target cells, with degranulation levels comparable to those of the ADCC-inducing antibody 10-1074 (Fig. 4c). In contrast, VRC01 failed to elicit any detectable NK cell degranulation under the same conditions (Fig. 4c). Additionally, intracellular staining revealed that FD22 significantly increased the production of IFN-γ and β-chemokines (e.g., MIP-1β) (Fig. 4c), key markers of NK cell activation. These findings highlight the dual functionality of FD22, which not only engages FcγRIIIa receptors to drive direct cytotoxic activity but also stimulates cytokine and chemokine production, effectively activating NK cells. This dual action underscores the potential of FD22 to facilitate robust ADCC against HIV-1-infected target cells.

To evaluate the ADCC induction activity of FD22, an LDH release cytotoxicity assay was conducted using HIV-1-infected H9 (IIIB) cells as target cells and PBMCs from healthy donors as effector cells. FD22 was tested at a series of concentrations (200, 100, 50, 25, 12.5, and 6.25 μg/mL) to determine its efficacy in inducing ADCC. The target and effector cells were co-incubated at an effector-to-target (E: T) cell ratio of 6:1. The results showed that FD22 induced robust ADCC in a concentration-dependent manner, achieving up to 80% cytotoxicity at the highest concentration (Fig. 4d). Notably, the ADCC induction activity of FD22 surpassed that of the well-characterized ADCC-inducing antibody 10-1074, especially at relatively high concentrations (Fig. 4d). In contrast, VRC01 exhibited very low ADCC induction activity under identical conditions (Fig. 4d), further emphasizing the superior effector function of FD22 in mediating immune cell activation and viral clearance. These findings highlight the dual functionality of FD22, which not only exhibits potent neutralizing activity against diverse HIV-1 strains but also enhances immune responses to eliminate infected cells. This dual capability underscores FD22’s potential as a therapeutic antibody.

### Structural prediction of the HIV Env SOSIP trimer–FD22 Fab interactions with AlphaFold3

**Fig. 5:**
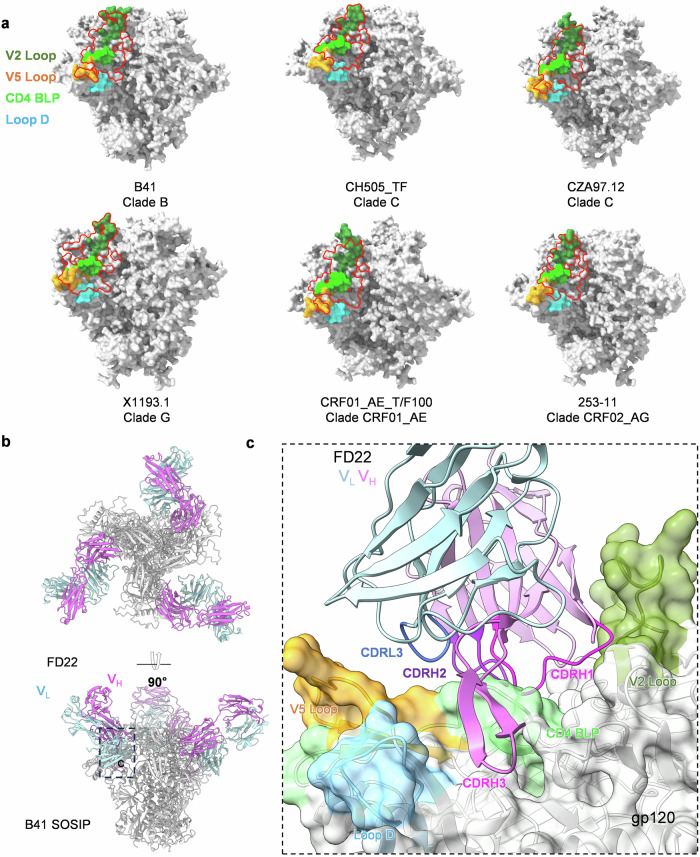
Structural prediction of the FD22 Fab-HIV Env SOSIP trimer complex with AlphaFold3. **a** Predicted binding epitopes of FD22 Fab on the HIV Env SOSIP trimer of multiple FD22-sensitive viruses by AlphaFold3. Four important regions of gp120, the V2 Loop, Loop D, the CD4 BLP, and the V5 Loop, are highlighted in different colors, and the epitopes are circled by red lines. **b** Predicted structure of the FD22 Fab–B41 Env SOSIP trimer complex. Top and side views of the predicted FD22 Fab–B41 Env SOSIP trimer complex. The B41 Env SOSIP trimer is shown in gray, with the FD22 heavy chain in orchid and the light chain in powder blue. ModelSeeds ranking score: 0.75; additional parameters are provided in Supplementary Table S3. **c** Interaction interface between FD22 and the B41 Env SOSIP trimer. Predicted binding interface between the B41 Env SOSIP trimer and the complementarity-determining regions (CDRs) of FD22, with individual CDRs highlighted in distinct colors.

On the basis of competitive antibody binding experiments, we initially identified FD22 as a CD4bs-targeting antibody. Given the availability of numerous high-resolution structures of CD4bs antibodies complexed with Env proteins from various HIV-1 strains in the PDB database and the accurate structure prediction capabilities of AlphaFold3^29^, we aimed to obtain detailed epitope information for FD22. To achieve this goal, we used AlphaFold3 to predict the interactions between the Fab fragment of FD22 and the Env SOSIP trimer of multiple FD22-sensitive virus strains. The template modeling (TM) scores for these predictions ranged from 0.6 to 0.8, a confidence range indicating that the structural predictions were partially accurate (confidence summary provided in Supplementary Table S2). We further analyzed the interaction interfaces between the FD22 Fab and the Env SOSIP^30^ complex by PDBePISA^31^ and observed several consistent features across the models. In all six virus strains sensitive to FD22, the interfaces were located in a conserved region of the gp120 outer domain. Specifically, these interfaces overlapped with key structural elements, including the V2 Loop, Loop D, the CD4 binding loop (CD4 BLP), and the V5 Loop (Fig. 5a). These findings provide valuable insights into the epitope specificity of FD22 and highlight its ability to target critical regions on the gp120 outer domain.

Using strain B41, which presented the highest ptm and iptm scores, as an example, we identified that the CDRs of the FD22 heavy chain constituted the primary interaction interface with Env (Fig. 5b, c). Specifically, CDRH1 was oriented primarily toward the V2 Loop, while CDRH2 and CDRH3 encircled the CD4 BLP. Notably, CDRH3 was also positioned adjacent to Loop D, further enhancing binding specificity. In addition to the heavy chain, the light chain contributed significantly to the interaction. The CDR3 of the light chain (CDRL3) was located near the V5 loop, playing a substantial role in the overall antigen–antibody engagement (Fig. 5c; Supplementary Tables S3 and S4). These structural details highlight the precise and multifaceted binding mechanism of FD22, involving both heavy and light chains in the targeting of critical regions of the Env protein.

**Fig. 6:**
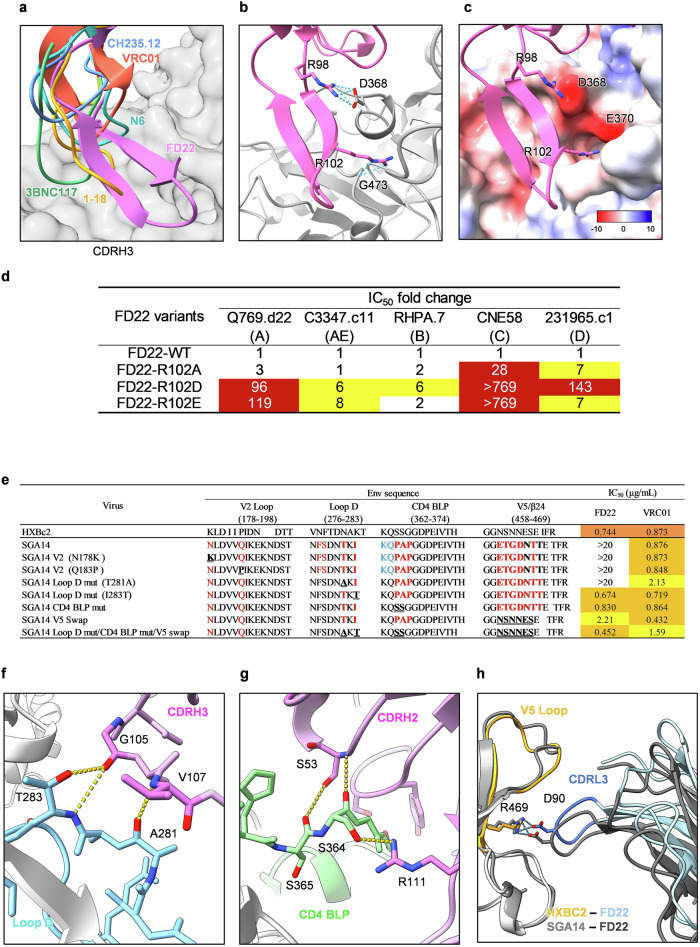
Structural analysis of the B41 Env SOSIP trimer–FD22 Fab complex. **a** Superimposition of CD4bs antibodies on gp120. Structural alignment of six CD4 binding site (CD4bs) antibodies in complex with gp120: VRC01 (PDB ID: 8VGW), CH235.12 (PDB ID: 8VH2), 1-18 (PDB ID: 6UDJ), 3BNC117 (PDB ID: 5V8M), and N6 (PDB ID: 5TE6). **b** Close-up of the interactions of CDRH3 with gp120. Detailed view of the interaction between FD22 CDRH3 and gp120, highlighting residues involved in hydrogen bonding. **c** Electrostatic surface potential of gp120. Surface electrostatic potential mapping of gp120, corresponding to the region shown in (**a**). **d** Neutralization activity of FD22 variants against pseudotyped viruses across different clades. Variants exhibiting reduced neutralization potency are color-coded based on the magnitude of change: red indicates >10-fold increase in IC_50_, while yellow represents a 5–10-fold increase. **e** Neutralization sensitivity of the resistant autologous virus SGA14 and its mutants to FD22. The resistant autologous virus SGA14, derived from P27, was engineered by substituting the V2 Loop, Loop D, CD4 BLP, and V5 Loop of HXBc2 Env into SGA14. Sequence variations in the P27 autologous virus relative to the HXBc2 reference are highlighted in red, with reverse mutations indicated in bold and underlined. IC_50_ values are represented using a color code: red (<0.1 μg/mL), orange (0.1–1 μg/mL), and yellow (>1 μg/mL). VRC01 was included as a control for comparative analysis. **f** Detailed view of FD22 Fab interactions with gp120 Loop D. Predicted interactions between FD22 Fab and the Loop D region of gp120, highlighting residues involved in hydrogen bonding. AlphaFold3 parameters: ModelSeeds = 1692646031, ranking score = 0.63. Additional details are provided in Supplementary Table S4. **g** Detailed view of FD22 Fab interactions with the CD4 BLP. Predicted structural interactions between FD22 Fab and the CD4 BLP of gp120, with residues forming hydrogen bonds highlighted. AlphaFold3 parameters: ModelSeeds = 1692646031, ranking score = 0.63. Additional details are provided in Supplementary Table S4. **h** Local superimposition of the predicted FD22 Fab-HXBc2 SOSIP and FD22 Fab-SGA14 SOSIP complexes. Structural alignment of the predicted FD22 Fab in complex with HXBc2 SOSIP and SGA14 SOSIP, showing residues involved in hydrogen bonding.

We compared the epitope profiles of FD22 with those of five other CD4bs-targeting antibodies (CH235.12, 3BNC117, 1-18, VRC01, and N6). As shown in Supplementary Fig. S3a, all antibodies target the core CD4 binding site; however, detailed analysis revealed unique structural features specific to FD22. Notably, FD22 possesses an unusually long CDRH3 loop, which includes a critical arginine residue (R102) that contributes to its distinct binding mode (Fig. 6a, b; Supplementary Fig. S3b). This positively charged residue facilitates electrostatic interactions with a negatively charged pocket on the gp120 surface, thereby enhancing binding specificity (Fig. 6c). To further validate the critical role of the R102 residue in the CDRH3 loop of FD22, we performed site-directed mutagenesis, substituting the positively charged arginine (R) with a non-polar residue alanine (R102A), and with negatively charged residues glutamic acid (R102E) and aspartic acid (R102D). Neutralization assays revealed that the R102A mutation significantly impaired neutralizing potency, resulting in 28-fold and 7-fold increases in IC_50_ against CNE58 and 231965.c1, respectively. Strikingly, both charge-reversal mutants, R102D and R102E, significantly reduced neutralizing activity across all tested strains. This effect was especially pronounced for CNE58, where the IC_50_ fold change exceeded 769-fold for both mutants (Fig. 6d). These results highlight the essential role of the electrostatic properties of R102 in mediating specific interactions with the CD4 binding site, and reveal a distinct mode of engagement that sets FD22 apart from other CD4bs antibodies.

### The neutralizing mechanism of FD22

Autologous viruses from donor P27 were cloned and constructed by reverse transcription followed by single-genome amplification (SGA). Clone SGA14 was found to be resistant to FD22 (Fig. 6e; Supplementary Table S5). To determine the contributions of these mutations to the resistance of FD22, SGA14 was reverse mutated to the sensitive HIV^HXBc2^ sequence at the V2 Loop, Loop D, the CD4 BLP, and the V5 Loop. Reverse mutations in the V2 Loop did not alter the sensitivity of FD22 (Fig. 6e; Supplementary Fig. S4). However, reversion mutations in Loop D, the CD4 BLP, and the V5 Loop all enhanced the neutralization activity of FD22. Notably, the I283T reverse mutation in Loop D significantly increased the sensitivity of SGA14 to FD22, yielding an IC_50_ of 0.674 μg/mL. Structural predictions indicated that T283 could form hydrogen bonds with CDRH3 (Fig. 6f). Similarly, mutations in the CD4 BLP and the V5 Loop (amino acids 458–469) improved FD22 neutralization sensitivity, yielding IC_50_ values of 0.830 μg/mL and 2.21 μg/mL, respectively (Fig. 6e; Supplementary Fig. S4). AlphaFold3 predictions indicate that the S364 and S365 residues in the CD4 BLP form multiple hydrogen bonds with FD22 (Fig. 6g), while the V5 Loop of the sensitive HIV^HXBc2^ strain is predicted to be positioned closer to the FD22 light chain (Fig. 6h). These findings not only reveal the epitope of FD22, but also validate the accuracy of AlphaFold3 in predicting the interaction mechanisms of FD22. These results provide valuable insights into the structural basis of FD22 neutralization, ADCC activity, and its potential for overcoming viral resistance.

## Discussion

China has a distinct prevalence of HIV-1 subtypes, with CRF01_AE and CRF07_BC being the dominant genotypes^32,33^. CRF01_AE impairs immune recovery, while CRF07_BC shows higher transmission and has a highly glycosylated V1 region that may hinder bNAb binding. Thus, identifying potent and broadly neutralizing CD4bs antibodies from Chinese patients could play a pivotal role in the development of effective HIV therapies and vaccines tailored to diverse populations. In this study, we isolated and characterized a broad and potent CD4bs neutralizing antibody FD22, which neutralized 82% of a panel of 145 diverse HIV-1 pseudoviruses, including strong activity against the major Chinese strains CRF01_AE and CRF07_BC. Interestingly, the heavy chain of FD22 was derived from the 3-30*4 germline, which differs from the more commonly identified germline alleles 1-2*02 or 1-46*01 frequently associated with potent breadth and neutralization. Notably, the CD4bs bNAb HJ16, also derived from the 3-30*4 germline, exhibited limited neutralization, targeting only 36% of the 94 tested pseudoviruses^34^. This suggests that while IGHV3-30*4 alleles may contribute to neutralization breadth, their functional outcomes depend on additional structural or genetic factors. Our findings highlight an alternative genetic pathway for generating CD4bs-directed bNAbs, expanding the repertoire of therapeutic and vaccine targets against HIV-1.

A key safety consideration for bNAbs is the potential for autoreactivity. To evaluate this risk, we performed standard preclinical assays, including HEp-2 cell staining and cardiolipin ELISA, both of which revealed no detectable autoreactivity for FD22. These findings suggest a favorable preliminary safety profile. However, the comprehensive preclinical evaluation, including studies on biodistribution, immunogenicity, and toxicity, is necessary to fully assess the in vivo safety and therapeutic potential of FD22.

In addition to the specific recognition of Fab, which blocks viral entry, the antiviral activity of bNAbs is also mediated by the Fc domain, which interacts with Fcγ receptors to activate distinct immunomodulatory pathways^35^. Notably, differences have been observed among bNAbs in their capacity to eliminate HIV-1-infected lymphocytes^36^. However, the necessity of Fc functions for bNAb efficacy remains debated. For instance, studies of PGT121 reported Fc-independent protection in macaque models^37–39^. In contrast, other bNAbs such as 10-1074, b12, and 3BNC117 have been shown to eliminate SHIV-infected cells or replication-competent viral reservoirs through Fc-mediated effector functions in vivo^40–43^. In this study, we used 10-1074 as a positive control for ADCC activity. We found that FD22 strongly binds to HIV-1-infected cell lines, induces FcγRIIIa activation in both HIV-infected and latently infected cells, and triggers NK cell degranulation and the release of key cytokines, reaching levels comparable to those induced by 10-1074 (Fig. 4a–c). Notably, results from the LDH release cytotoxicity assay revealed that FD22 induced robust ADCC in a concentration-dependent manner, achieving up to 80% cytotoxicity at the highest concentration, surpassing both VRC01 and the ADCC-inducing antibody 10-1074 (Fig. 4d). This underscores the critical role of FD22’s Fc function in mediating immune clearance of infected cells. FD22’s dual functionality — simultaneously neutralizing HIV-1 and activating ADCC — positions it as a next-generation therapeutic candidate.

FD22 does not compete with 3BNC117 or 1-18 in competition assay, suggesting a distinct mode of binding. Structural analysis of these three antibodies in complex with the HIV Env SOSIP trimer (PDB: 6UDJ, 5V8M; see Supplementary Fig. S3) reveals critical mechanistic differences. 1-18 and 3BNC117 employ a dual-epitope binding mode: (1) targeting the canonical CD4 binding site and (2) engaging adjacent gp120 protomers via CDRH1 loop interactions (1-18) or framework region 1 (FR1) contacts (3BNC117) (Supplementary Fig. S3a). In contrast, FD22 lacks these structural features. FD22 features shorter CDRH1 and FR1 loops, which are structurally incapable of mediating such inter-protomer interactions. These mechanistic differences explain the unique competition profile of FD22 and highlight its distinct strategy for targeting the CD4bs.

Structural analysis of the FD22–Env complex highlights the importance of interactions mediated by Loop D, the CD4 BLP, and the V5 loop of Env, alongside the unusually long CDRH3 loop of FD22. Notably, the positively charged residue R102 at the apex of this CDRH3 loop facilitates direct engagement with Env’s conserved regions, driving both potent neutralization and FcγRIIIa-dependent ADCC. The absence of autoreactivity observed with FD22 further underscores its potential safety as a therapeutic candidate. Future studies are needed to evaluate the in vivo efficacy of FD22 in animal models and to explore its potential use in combination with antibody therapies or as a blueprint for vaccine design.

In summary, we identified and characterized FD22, a novel CD4bs antibody isolated from a Chinese HIV-1 elite controller. FD22 exhibited potent neutralizing activity and strong ADCC function, with structural analyses revealing key interactions responsible for its neutralization mechanism. These findings highlight FD22 as a promising candidate for effective HIV-1 therapy.

## Materials and methods

### Cell lines

The following cell lines were used: U87 CD4^+^CCR5^+^, ACH-2, HL2/3, H9 (IIIB), HEp-2, and TZM-bl cells, which were obtained from the NIH AIDS Program; HEK293F cells from Sino Biological; HEK293T cells from the National Collection of Authenticated Cell Cultures; and Jurkat-Lucia NFAT-CD16a cells from InvivoGen. U87 CD4^+^CCR5^+^ cells were cultured in DMEM (HyClone) supplemented with 15% FBS (Gibco), Penicillin-Streptomycin (Cytiva), and 1× Biomyc-3 (Biological Industries) at 37 °C and 5% CO_2_. ACH-2 and H9 (IIIB) cells were maintained in RPMI 1640 medium with 10% FBS, Penicillin-Streptomycin (Cytiva), and Biomyc-3 (Biological Industries) at the same temperature and CO_2_ levels. HEK293T, HL2/3, and HEp-2 cells were cultured in DMEM containing 10% FBS, Penicillin-Streptomycin (Cytiva), and Biomyc-3 (Biological Industries) at 37 °C and 5% CO_2_. HEK293F cells were kept in SMM 293-TII Medium (Sino Biological Inc.) under identical environmental conditions, with shaking at 120 rpm.

### Study participants

HIV-1 patients were recruited through the Xuchang Center for Disease Control and Prevention (Henan Province, China). Ethical approval for this study was granted by the Ethics Committee of the School of Basic Medical Sciences, Fudan University (Approval No. 2017-C0071). Written informed consent was obtained from all participants, ensuring their voluntary participation and compliance with ethical guidelines.

Donor P27 was selected for B-cell isolation and antibody development due to the serum’s exceptionally broad and potent neutralizing activity, which stood out among all participants in the study. This donor was classified as a slow progressor, having lived with HIV-1 for 23 years without requiring antiretroviral therapy at the time of leukapheresis. Donor P27’s unique clinical profile made him an ideal candidate for investigating broadly neutralizing antibodies and their potential for therapeutic development.

### Memory B-cell isolation and antibody production

Memory B cells were processed and sorted following an established protocol^21^. Briefly, peripheral blood-derived switch-memory B cells were sorted into 384-well plates, with four cells placed in each well. These cells were then stimulated and cultured for 13 days using IL-2, IL-21, and 3T3-msCD40L feeder cells. Supernatants exhibiting neutralizing activity were selected, and the variable regions of the heavy and light chains of the immunoglobulin genes were amplified using RT-PCR. These genes were subsequently cloned and re-expressed according to previously described methods.

### Pseudovirus generation

HIV-1 Env pseudoviruses were generated by co-transfecting 70%–80% confluent HEK293T cells with a backbone plasmid lacking the Env gene (pNL4-3.Luc.R-E-) and a plasmid encoding the HIV-1 Env protein, using EZ Trans reagent (Life-iLab, China) at a 4:1 ratio. Culture supernatants containing pseudoviruses were harvested 48 h post-transfection and stored at –80 °C. For generating pseudovirus mutants, specific mutations were introduced into the HIV Env expression plasmid using site-directed mutagenesis PCR, primers were designed with the QuikChange® Primer Design Program. Mutant pseudoviruses were then produced using the same procedure as for wild-type pseudoviruses.

### Neutralization experiments

The neutralizing capacity of monoclonal antibodies and serum samples was evaluated using a single-round HIV-1 Env-pseudovirus infection assay. U87 CD4^+^CCR5^+^ cells expressing the CD4 receptor and CCR5 co-receptor were used as target cells. Heat-inactivated serum or monoclonal antibodies were serially diluted (5-fold) and incubated with the pseudovirus for 30 min. The mixture was then added to U87 CD4^+^CCR5^+^ cells and incubated for 48 h. Afterward, a luciferase assay system (Promega) was used to measure infection, and relative light units (RLU) were recorded using a luminometer (Perkin Elmer). ID_50_ and ID_80_ were defined as the serum dilution at which 50% and 80% of the virus is neutralized, respectively. IC_50_ values refer to the antibody concentration required to achieve 50% neutralization.

### HIV-1 envelope protein binding assays

The interaction between antibodies and gp120 or gp41 proteins was evaluated using ELISA. Antigens (2 μg/mL) were coated onto 96-well plates and incubated overnight at 4 °C. The plates were blocked with BLOTTO buffer (PBS with 1% FBS and 5% non-fat milk) for 1 h at room temperature. Afterward, antibodies were serially diluted in disruption buffer (PBS containing 5% FBS, 2% BSA, and 1% Tween-20) and incubated for 1 h. Plates were washed between steps with PBST (PBS containing 0.2% Tween-20). HRP-conjugated goat anti-human IgG antibody (diluted 1:2500) was added and incubated for 1 h at room temperature. After a final wash, ABTS substrate was applied, and absorbance was measured at 405 nm. Each ELISA was repeated at least twice, with representative data presented.

### Competitive ELISA assays

To assess antibody competition, target antibodies were biotinylated using EZ-Link NHS-LC-Biotin (Thermo Fisher), following the manufacturer’s instructions. Buffer exchange was performed with 10 kDa centrifugation filter membranes (Millipore) for stability. Plates were coated overnight at 4 °C with HIV^JRCSF.JB^ gp120 protein (2 μg/mL) and blocked with 2% BSA in PBS for 2 h at room temperature. After blocking, the plates were washed three times with PBST (PBS containing 0.05% Tween-20). Serial dilutions of competitor antibodies (50 µL) were added, followed by a 2 h incubation. Subsequently, 50 µL of FD22-biotin (1 μg/mL final concentration) was added to the wells and incubated for 30 min. Plates were then washed five times, and streptavidin-HRP (diluted 1:1000) was added, followed by incubation for 1 h at room temperature. ABTS substrate was then applied, and absorbance was recorded at 405 nm. The inhibition of FD22-biotin binding to gp120^JRCSF.JB^ was quantified by comparing the area under the curve (AUC) with that of a non-gp120-binding isotype control antibody.

### Biolayer Interferometry (BLI)

BLI measurements were conducted using an Octet RED96 system (ForteBio) with agitation set at 1000 rpm. Experiments were performed at 30 °C in black, solid 96-well plates, with all solutions prepared to a final volume of 200 μL per well in 1× kinetic buffer^44^. For evaluating the *K*D of antibody–gp120 interactions, AHC biosensors were used to load antibodies at concentration of 10 μg/mL in kinetic buffer (1× PBS, pH 7.4 with 0.2% Tween-20). Binding of gp120 was monitored using a dilution series ranging from 900 nM to 11.1 nM. Data were analyzed with ForteBio’s acquisition software (version 8.1) and fitted using a 1:1 global model to determine the *K*D values.

### Cell surface binding assays

To investigate antibody binding to cells expressing the HIV envelope or infected with HIV, cells were cultured for 48 h before the experiment. For ACH-2 cells, a model of latent HIV infection, latent viruses were reactivated by treating cells with TNF-α for 48 h prior to testing^45^. H9 (IIIB) cells, ACH-2 cells, and HL2/3 cells were incubated at 37 °C for 1 h with bNAbs or a control antibody (human IgG1), each at a concentration of 12.5 μg/mL in PBS containing 2% FBS. After incubation, cells were washed and stained with a 1:1000 dilution of Live/Dead Dye (Thermo Fisher) and a 1:200 dilution of Alexa Fluor 647-conjugated goat anti-human IgG (H + L) antibody (Thermo Fisher). Following staining, cells were resuspended in PBS containing 1% FBS, and antibody binding was analyzed by flow cytometry.

### Inhibition of reactivated HIV-1 latently infected cells

Latent HIV-1-infected ACH-2 cells were reactivated using 10 ng/mL TNF-α or 1 μM panobinostat for 48 h, and reactivation levels were quantified through P24 ELISA. Virus-containing supernatants from reactivated cells were collected and incubated with serial 3-fold dilutions of bNAbs at 37 °C for 30 min. The mixture of antibody and activated virus was then added to TZM-bl cells pre-seeded in 96-well plates. After 12 h, supernatants were replaced with fresh DMEM supplemented with 10% FBS to eliminate residual virus and prevent reinfection. Cells were incubated for another 48 h, then lysed. A portion of the cell lysate (30 μL) was transferred to a white microtiter plate (Corning), and luminescence was measured after adding the substrate (Promega) using a luminometer (Perkin Elmer).

### P24 ELISA

ACH-2 cells, representing a model of latent HIV infection, were treated with TNF-α or panobinostat to induce viral reactivation. Supernatants from activated cultures were mixed 1:1 with 5% Triton X-100 and incubated overnight to lyse cells. HIV-1 p24 antigen levels were quantified using a sandwich ELISA, calibrated against a standard curve generated with recombinant p24 protein ranging from 100 ng/mL to 0.078 ng/mL. Rabbit polyclonal anti-Gag-p24 antibodies (Sino Biological Inc.) were used to coat ELISA plates. After blocking, supernatants from reactivated cells were added, followed by mouse anti-P24 antibody 183-12H-5C. Detection was carried out with HRP-conjugated horse anti-mouse IgG (Cell Signaling). ABTS solution (Thermo Fisher) was used for color development, and absorbance was recorded at 405 nm. Negative controls included untreated cell culture medium.

### FcγRIIIa stimulation assay

We evaluated the ADCC activity of FD22 by assessing its ability to induce signaling through FcγRIIIa (CD16a), the primary receptor expressed on NK cells. We utilized the Jurkat-Lucia NFAT-CD16a cell line to assess the activation of nuclear factor of activated T cells (NFAT cells), an early signaling event in ADCC induction^28^. The level of ADCC induction was determined by measuring the luciferase signal generated from an NFAT-dependent reporter protein. HIV-1-infected cell line H9 (IIIB), HL2/3, and HIV-1 reservoir-reactivated cell line ACH-2 were incubated with the bNAbs. Jurkat-Lucia NFAT-CD16a cells were then added and co-cultivated with the target cells for 24 h at 37 °C. After incubation, the Jurkat-Lucia NFAT-CD16a cells were transferred to a white 96-well half-plate, and luciferase activity was developed using the Nano-Glo Live Cell Reagent (Promega, cat: N2058). The RLU values were measured using an EnSight plate reader (Perkin Elmer). The fold change in luciferase gene expression relative to the no-antibody control was calculated.

### NK cell activation

PBMCs from healthy donors were isolated using Ficoll density gradient centrifugation. NK cells were then enriched from the PBMCs using the NK Cell Isolation Kit (Miltenyi Biotec, cat: 130-092-657). Prior to in vitro activation assays, NK cells were rested and subsequently activated by incubation with recombinant human IL-15 (1 ng/mL) (Gibco, cat: PHC9154) in IMEM medium (Gibco, cat: 12440053) for 12 h.

For the NK cell activation and cytokine secretion assay, H9 (IIIB) cells were used as target cells and NK cells as effector cells at an effector-to-target (E: T) ratio of 20:1. Briefly, 1 × 10^4^ H9 (IIIB) cells were pre-incubated with 200 μg/mL of the indicated antibody for 30 min in a cell culture incubator. Subsequently, 2 × 10^5^ NK cells were added. To assess NK cell activation, 20 μL of mouse anti-human CD107a-PE (BD Pharmingen, cat: 555801) was added to each well. For cytokine detection, the protein transport inhibitor BFA (10 μg/mL) was added, and cells were incubated for 6 h. Following incubation, cells were stained with fluorescently conjugated antibodies: mouse anti-human CD3-PerCP-Cy5.5 (BD Pharmingen, cat: 560835), mouse anti-human CD56-PE-Cy™7 (BD Pharmingen, cat: 557747), and mouse anti-human CD16-FITC (BD Pharmingen, cat: 555406). After surface marker staining, cells were fixed and permeabilized using the Fixation/Permeabilization Solution Kit (BD Biosciences, cat: 554714) and then stained with mouse anti-human IFN-γ (BD Pharmingen, cat: 564791) and mouse anti-human MIP-1β (BD Pharmingen, cat: 560686). NK cells were identified as CD3 negative cells. NK cell activation was determined by measuring the expression of CD107a, IFN-γ, and MIP-1β using flow cytometry.

### ADCC evaluation

We evaluated ADCC of FD22 against HIV-1-infected target cells using the H9 (IIIB) cell line as the target and PBMCs from healthy donors as effector cells. ADCC activity was measured using the CytoTox 96 Non-Radioactive Cytotoxicity Assay (Promega, cat: G1780), following previously described protocols^46,47^. Briefly, 8 × 10^4^ H9 (IIIB) cells were incubated with 25 μL of serially diluted monoclonal antibody (mAb) in a U-bottom tissue-treated 96-well plate for 30 min in a cell incubator. Next, 4.8 × 10^5^ PBMCs, cultured with recombinant human IL-15 (1 ng/mL), were added to each well to achieve an effector-to-target (E: T) ratio of 6:1. The plate was spun at 300× *g* for 5 min to facilitate effector-target cell contact and then incubated for 40 h. Spontaneous background LDH release from target and effector cells was assessed by replacing the mAb with medium. Maximum LDH release was determined by adding lysis solution (Promega, cat: G182A) to wells with only target cells 45 min before detection. After incubation, the plate was spun at 300× *g* for 5 min, and 50 μL of supernatant was transferred to a new transparent flat-bottom plate. CytoTox 96 reagent (Promega, cat: G179A) was added, followed by a 30 min incubation at room temperature in the dark. The reaction was stopped with 50 μL of stop solution (Promega, cat: G183A), and absorbance was measured at 490 nm. ADCC activity was calculated as follows: Cytotoxicity (%) = [(OD_490_ of mAb treated − OD_490_ of no mAb control)/(OD_490_ of H9 (IIIB) lysate − OD_490_ of H9 (IIIB) cells only)] × 100.

### Autoreactivity assays

Reactivity of FD22 to HIV-1-negative human epithelial (HEp-2) cells was evaluated by indirect immunofluorescence. Slides were prepared and stained with Alexa Fluor 647-conjugated goat anti-human IgG (H + L) (Invitrogen) following standard protocols. Imaging was performed using a Leica fluorescence microscope, and photographs were captured with a 15 s exposure for each mAb to assess binding to HEp-2 cells.

### Polyreactivity assay

Ployreactivity of FD22 to Cardiolipin was evaluated as previously described^23^. A 96-well flat-bottom MaxiSorp plate was coated with cardiolipin (20 μg/mL in ethanol) and incubated overnight at 4 °C. The plate was then blocked with 300 μL/well of 3% BSA in PBS for 2 h at room temperature (RT). Antibodies were serially diluted in 3-fold steps using PBS-Tween-20 (0.05%) containing 3% BSA and 2% FBS, and 100 μL of each diluted antibody solution was added to the wells. The plate was incubated for 2 h at RT. After incubation, the plate was washed five times with PBS containing 0.2% Tween-20. Subsequently, 100 μL/well of HRP-conjugated goat anti-human IgG (diluted 1:2500; Jackson ImmunoResearch) was added and incubated for 1 h at RT. Following this, the plate was washed again five times with PBS containing 0.2% Tween-20. Finally, the assay was developed using ABTS (Thermo) as the substrate, and the absorbance was measured at 405 nm to determine antibody reactivity.

### Single-genome amplification (SGA)

SGA of the HIV-1 env gene from patient P27 was conducted as previously reported^48^. The cDNA was serially diluted to identify a dilution with approximately 30% PCR-positive reactions, ensuring that most positive wells contained amplicons from SGA single cDNA molecules. The Platinum Taq High Fidelity system (Invitrogen) was used for PCR of the full-length envelope gene, with 20 μL reaction volumes containing 2 μL 10× buffer, 0.8 μL MgSO_4_, 0.4 μL dNTPs, 0.4 μL of each primer (2 μM), 0.2 μL Taq polymerase, and 1 μL template DNA. For the initial round of PCR, EnvB5out (5′-TAGAGCCCTGGAAGCATCCAGGAAG-3′) and EnvB3out (5′-TTGCTACTTGTGATTGCTCCATGT-3′) primers were employed, while EnvB5in (5′-CACCTTAGGCATCTCCTATGGCAGGAAGAAG-3′) and EnvB3in (5′-GTCTCGAGATACTGCTCCCACCC-3′) were used for the nested PCR. The PCR conditions were 94 °C for 2 min, followed by 40 cycles of 94 °C for 15 s, 55 °C for 30 s, and 68 °C for 4 min, concluding with 68 °C for 10 min. Second-round amplification followed similar conditions but included 45 cycles. Products were checked on 1% agarose gels. PCRs were conducted in a contamination-controlled workspace.

### DNA sequencing

Amplicons were sequenced at BGI Tech Solutions (Beijing Liuhe) CO., Ltd, with both DNA strands analyzed through partially overlapping regions. The resulting sequence fragments for each amplicon were assembled and refined using DNASTAR 11.0. Chromatograms were carefully reviewed to identify any mixed base sites (double peaks), which could indicate the use of multiple templates during priming or errors introduced in early PCR cycles. Sequences exhibiting double peaks were excluded from further evaluation to ensure accuracy.

### Cloning of HIV-1 env genes from donor P27

Selected env gene sequences from donor P27 were cloned for further study. Full-length env and rev genes were amplified from second-round PCR products using Primestar Polymerase (TAKARA) and specific primers: HIV RevS NheI (5′-GCTCGCTAGCACCATGGCAGGAAGAAG-3′) and HIV EnvR XhoI (5′-GCTACTCGAGTTATAGCAAAGCCCTTTCGAG-3′). The resulting PCR products were purified from gels and inserted into the pcDNA3.1(-) expression vector (Invitrogen Life Technologies) under the control of a T7 promoter. These plasmids were then transformed into TOP10 chemically competent E. coli cells. Plasmid DNA was extracted using the AXYGEN Plasmid Miniprep Kit, and the sequence of each plasmid was verified. All four cloned env sequences from donor P27 were confirmed to be functional, mediating viral entry with 100% success.

### Prediction and analysis of FD22-HIV Env SOSIP complex by AlphaFold3

The 3D structure of the FD22 Fab and its complex with HIV Env (SOSIP) was predicted using AlphaFold3 (https://alphafoldserver.com/)^29^, a state-of-the-art deep learning model for protein structure prediction. The AlphaFold network was employed to predict the 3D coordinates of all heavy atoms of the FD22 Fab and the FD22-SOSIP complex. Multiple seeds were used to generate different conformations of the antigen–antibody complex, with subsequent ranking of predictions to improve the overall accuracy and reliability of the structural model. The confidence level of the model predictions was assessed, and the details are presented in Supplementary Table S4. The predicted models were subjected to further structural analysis to identify key interactions between the FD22 Fab and HIV Env, using the PDBePISA web server (https://www.ebi.ac.uk/pdbe/pisa/)^31^. This tool allowed for the identification and analysis of protein–protein interactions, including the determination of interaction interfaces, binding energy, and key residues involved in the complex formation.

For visualization and further structural interpretation, the ChimeraX software was utilized. ChimeraX enabled the detailed imaging and analysis of the predicted complex, providing insights into the structural arrangement of FD22 binding to HIV Env. This approach allows us to examine the molecular basis of the antibody-antigen interaction and gain a better understanding of how FD22 targets HIV Env for potential therapeutic applications.

## Supplementary information

Supplementary Information
