# Supplementary material for: A potent and broad CD4 binding site neutralizing antibody with strong ADCC activity from a Chinese HIV-1 elite neutralizer
Source: Cell Discov. 2025 Jun 10;11:55. doi: 10.1038/s41421-025-00808-x (PMC12149299; doi:10.1038/s41421-025-00808-x)
Supplement: Supplementary file 1 — Supplementary Information [file 41421_2025_808_MOESM1_ESM.pdf]

Supplementary Materials for

A Potent and Broad CD4 Binding Site Neutralizing Antibody with Strong ADCC Activity from a Chinese HIV-1 Elite Neutralizer

Yingdan Wang[1,2#], Ping Ji[1#], Qianying Liu[3#], Nannan Jia[2#], Yunping Ma[1], Tianyi Yuan[4], Palizhati Rehati[1], Jiali Chen[1], Yumei Wen[1], Fan Wu[2]*, Jinghe Huang[1]*

[1]Key Laboratory of Medical Molecular Virology (MOE/NHC/CAMS) and Shanghai Institute of Infectious Disease and Biosecurity, Shanghai Frontiers Science Center of Pathogenic Microorganisms and Infection, School of Basic Medical Sciences, Fudan University, Shanghai, China.

[2]Shanghai Immune Therapy Institute, Shanghai Jiao Tong University School of Medicine Affiliated Renji Hospital, Shanghai, China.

[3]School of Basic Medical Sciences, Southwest Medical University, Luzhou, Sichuan, China

[4]Xuchang center for disease control and prevention, China

[#]These authors contributed equally: Yingdan Wang, Ping Ji, Qianying Liu, Nannan Jia,

*Correspondence: Fan Wu (wufan@renji.com) and Jinghe Huang (Jinghehuang@fudan.edu.cn)

Keywords: HIV; Neutralizing antibody; CD4 binding site; ADCC

Supplementary Table S1. Antibody neutralization data for 145 HIV-1 Env pseudoviruses.

No.	Virus ID	Clade	IC$_{50}$ (μg/mL)	
			FD22	VRC01
1	UG037.8	A	>20	0.139
2	RW020.2	A	0.243	0.148
3	Q842.d12	A	8.473	0.045
4	Q769.h5	A	0.841	0.069
5	Q769.d22	A	0.086	0.061
6	Q259.17	A	>20	0.184
7	Q168.a2	A	0.233	0.260
8	MS208.A1	A	0.729	0.135
9	MI369.A5	A	>20	0.415
10	MB539.2B7	A	0.176	0.431
11	MB201.A1	A	0.043	0.127
12	KER2018.11	A	0.352	1.189
13	BS208.B1	A	0.191	0.066
14	BI369.9A	A	4.832	0.263
15	BB539.2B13	A	2.588	0.150
16	BB201.B42	A	1.937	0.225
17	398-F1_F6_20	A	>20	0.271
18	3718.v3.c11	A	>20	0.192
19	3415.v1.c1	A	0.271	0.065
20	0439.v5.c1	A	>20	0.768
21	0260.v5.c36	A	1.068	0.087
22	p246F3 env	AC	3.210	0.066
23	6540.v4.c1	AC	>20	>20
24	3589.V1.C4	AC	0.254	0.130
25	3301.V1.C24	AC	0.335	0.154
26	0815.V3.C3	AC	0.228	0.031
27	TH966.8	AE	0.778	0.893
28	R3265.c6	AE	0.237	0.206
29	R2184.c4	AE	0.089	0.054
30	R1166.c1	AE	>20	0.910
31	pCNE8 env	AE	0.719	1.256
32	CNE55	AE	2.093	0.821
33	CNE5	AE	0.509	0.089
34	C4118.09	AE	0.102	0.155
35	C3347.c11	AE	0.098	0.097
36	C2101.c1	AE	0.121	0.223
37	620345.c1	AE	6.271	>20
38	T280-5	AG	0.409	0.058
39	T278-50	AG	0.202	>20
40	T266-60	AG	>20	0.317
41	T257-31	AG	0.196	0.745
42	T251-18	AG	>20	2.319
43	CRF02_AG clone 33	AG	0.167	0.028
44	CRF02_AG clone 278	AG	0.578	>20
45	CRF02_AG clone 266	AG	>20	0.734
46	CRF02_AG clone 255	AG	0.174	0.299
47	271-11	AG	1.326	0.198
48	242-14	AG	>20	>20
49	YU2.DG	B	0.037	0.085
50	WITO.33	B	>20	0.259
51	TRO.11	B	0.379	0.447
52	TRJO.58	B	1.085	0.334
53	THRO.18	B	0.882	1.941
54	SS1196.01	B	0.614	0.946
55	SF162.LS	B	0.076	0.255
56	SC422.8	B	0.867	0.223
57	RHPA.7	B	0.071	0.127
58	PVO.04	B	0.228	1.3880
59	pTRJO4551clone 58	B	1.059	0.139
60	JRFL.JB	B	0.231	0.058
61	JRCSF.JB	B	0.958	0.277
62	HO86.8	B	0.305	0.190
63	CNE64	B	>20	>20
64	CNE6	B	0.335	>20
65	CNE4	B	0.631	0.904
66	CNE12	B	0.075	0.063
67	CNE11	B	1.090	0.362
68	CNE10	B	0.243	0.485
69	CAAN.A2	B	2.362	1.654
70	BR07.DG	B	>20	2.438
71	BG1168.01	B	0.302	0.204
72	Bal.26	B	0.046	0.615
73	Bal.01	B	0.040	0.184
74	AC10.29	B	1.503	2.595

No.	Virus ID	Clade	IC_{50} (μg/mL)	
			FD22	VRC01
75	7165.18	B	2.237	>20
76	6535.3	B	0.130	1.359
77	6101.1	B	0.255	0.137
78	5768.04	B	>20	0.436
79	3988.25	B	>20	0.578
80	pBJOX2000 env	BC	>20	>20
81	CNE68	BC	0.909	0.272
82	CNE47	BC	0.285	3.976
83	CNE40	BC	0.134	0.286
84	CNE20	BC	0.165	>20
85	CNE16	BC	4.768	0.223
86	CNE15	BC	0.058	0.130
87	CH181.12	BC	0.556	0.752
88	ZM233.6	C	0.300	0.338
89	ZM215.8	C	0.288	0.298
90	ZM106.9	C	0.049	0.202
91	TV1.29	C	1.284	>20
92	pCE0217 env	C	0.081	0.195
93	DU422.01	C	16.230	>20
94	DU172.17	C	0.248	>20
95	DU156.12	C	>20	3.579
96	DU151.02	C	0.340	1.037
97	DU123.06	C	0.030	0.432
98	CNE66	C	>20	>20
99	CNE58	C	0.026	0.256
100	CNE56	C	>20	1.269
101	CNE53	C	3.459	0.136
102	CNE31	C	0.691	0.722
103	CNE30	C	8.889	1.084
104	CNE23	C	>20	15.140
105	CAP45.G3	C	5.689	1.909
106	CAP244.D3	C	0.075	0.471
107	CAP210.2.00.E8	C	>20	>20
108	BR025.9	C	0.038	0.176
109	96ZM651.02	C	0.684	1.782
110	6838.V1.C35	C	3.029	0.162
111	6644.V2.C33	C	0.080	0.197
112	6631.V3.C10	C	3.072	>20
113	3873.V1.C24	C	0.139	1.012
114	26191-2.48	C	0.023	0.184
115	25925-2.22	C	1.068	0.606
116	25711-2.4	C	0.066	0.270
117	25710-2.43	C	0.063	0.180
118	16936-2.21	C	0.413	0.137
119	16845-2.22	C	0.051	0.663
120	16055-2.3	C	2.062	0.141
121	0921.V2.C14	C	0.002	0.068
122	00836-2.5	C	0.007	0.025
123	0077_V1.C16	C	0.009	0.360
124	001428-2.42	C	0.322	0.017
125	286.36	C	0.003	0.185
126	SO18.18	C	0.006	0.063
127	TZBD.02	C	0.009	0.070
128	ZM109.4	C	0.043	0.065
129	ZM176.66	C	0.007	0.408
130	ZM197.7	C	0.295	0.010
131	ZM214.15	C	0.014	0.120
132	ZM53.12	C	4.827	0.468
133	ZM55.28a	C	0.080	0.029
134	QB099.391M.ENV.B1	C	0.060	0.046
135	3326.V4.C3	CD	0.017	0.007
136	3337.V2.C6	CD	>20	0.092
137	QD435.100M.ENV.E1	D	0.239	0.464
138	A03349M1.vrc4a	D	10.940	0.388
139	57128.vrc15	D	3.038	>20
140	231965.c1	D	0.011	0.019
141	247-23	D	>20	0.397
142	3016.v5.c45	D	0.252	0.081
143	6405.v4.c34	D	>20	0.902
144	X2088.c9	G	0.540	>20
145	pX1632 env	G	0.321	0.175
		GM IC_{50} (μg/mL)	0.27	0.25
		Median IC_{50} (μg/mL)	0.27	0.22
		Breadth (%)	82	88

IC_{50} values <0.1 μg/ml are highlighted in red, values between 0.1 and 1 μg/ml are highlighted in orange, and values between 1 and 20 μg/ml are highlighted in yellow.

Supplementary Table S2. Confidence summary of the predicted FD22-HIV Env SOSIP trimer complex structures.

Virus Strains	unit	fraction_disordered	has_clash	iptm	ptm
B41	3Fab+SOSIP[1]	0.04	0.0	0.72	0.76
	3Fab+SOSIP[2]	0.04	0.0	0.60	0.65
	Fab+gp120	0.04	0.0	0.76	0.77
CH505_TF	3Fab+SOSIP	0.02	0.0	0.63	0.67
	Fab+gp120	0.02	0.0	0.80	0.80
CZA97.12	3Fab+SOSIP	0.02	0.0	0.60	0.64
	Fab+gp120	0.02	0.0	0.79	0.79
X1193.1	3Fab+SOSIP	0.03	0.0	0.61	0.65
	Fab+gp120	0.03	0.0	0.79	0.8
CRF01_AE_T/E100	3Fab+SOSIP	0.04	0.0	0.67	0.71
	Fab+gp120	0.04	0.0	0.8	0.8
253-11	3Fab+SOSIP	0.04	0.0	0.57	0.61
	Fab+gp120	0.04	0.0	0.75	0.76
HXBc2	3Fab+SOSIP	0.03	0.0	0.66	0.70
	Fab+gp120	0.03	0.0	0.77	0.78

1: In AlphaFold3, ModelSeeds is 1, and ranking score is 0.75

2: In AlphaFold3, ModelSeeds is 1692646031, and ranking score is 0.63

Supplementary Table S3. Protein–protein interactions of the predicted FD22–B41 SOSIP trimer complex structure 1, analyzed by PDBePISA (ModelSeeds = 1, ranking score = 0.75)

Hydrogens bonds between B41 gp120 and FD22

##	B41-gp120	gp120 domain	Dist. [Å]	FD22	FD22 domain
1	A:LYS 178[NZ]	V2 Loop	2.85	B:ASP 73[OD2]	VH-FR3
2	A:LYS 178[O]	V2 Loop	2.19	B:TYR 75[OH]	VH-FR3
3	A:VAL 181[O]	V2 Loop	2.27	B:TYR 75[OH]	VH-FR3
4	A:PRO 183[O]	V2 Loop	2.91	B:GLY 26[N]	CDRH1
5	A:GLU 185[OE2]	V2 Loop	3.42	B:THR 3[OG1]	VH-FR1
6	A:THR 196[O]	V2 Loop	3.27	B:ARG 23[NH1]	VH-FR1
7	A:ASN 362[ND2]	CD4 BLP	3.68	C:ASP 90[OD2]	CDRL3
8	A:GLY 366[O]	CD4 BLP	2.87	B:LEU 100[N]	CDRH3
9	A:GLY 367[N]	CD4 BLP	3.00	B:SER 31[O]	CDRH1
10	A:GLY 367[O]	CD4 BLP	2.57	B:SER 31[OG]	CDRH1
11	A:ASP 368[N]	CD4 BLP	2.74	B:LEU 100[O]	CDRH3
12	A:ASP 368[OD1]	CD4 BLP	2.53	B:TYR 32[OH]	CDRH1
13	A:ASP 368[OD1]	CD4 BLP	2.61	B:ARG 98[NH1]	CDRH3
14	A:ASP 368[OD2]	CD4 BLP	2.98	B:ARG 98[NH2]	CDRH3
15	A:GLU 370[OE2]	CD4 BLP	2.74	B:ARG 102[NH1]	CDRH3
16	A:ARG 419[NH1]	β19	2.89	B:ASP 74[OD1]	VH-FR3
17	A:ARG 419[NH2]	β19	2.53	B:ASP 74[OD2]	VH-FR3
18	A:MET 426[O]	β20/β21	3.48	B:ARG 102[NH1]	CDRH3
19	A:ARG 469[NH2]	V5 Loop/β24	2.76	C:ASP 90[OD1]	CDRL3
20	A:ARG 469[NH1]	V5 Loop/β24	3.10	C:ASP 90[OD2]	CDRL3
21	A:GLY 473[O]	V5 Loop/β24	2.72	B:ARG 102[NE]	CDRH3
22	A:ARG 476[NH1]	V5 Loop/β24	3.71	B:GLY 105[O]	CDRH3

Salt bridges between B41 gp120 and FD22

##	B41-gp120	gp120 domain	Dist. [Å]	FD22	FD22 domain
1	A:LYS 178[NZ]	V2 Loop	2.85	B:ASP 73[OD2]	VH-FR3
2	A:LYS 178[NZ]	V2 Loop	3.77	B:ASP 74[OD2]	VH-FR3
3	A:ASP 368[OD1]	CD4 BLP	2.61	B:ARG 98[NH1]	CDRH3
4	A:ASP 368[OD2]	CD4 BLP	3.44	B:ARG 98[NH1]	CDRH3
5	A:ASP 368[OD1]	CD4 BLP	3.38	B:ARG 98[NH2]	CDRH3
6	A:ASP 368[OD2]	CD4 BLP	2.98	B:ARG 98[NH2]	CDRH3
7	A:GLU 370[OE2]	CD4 BLP	2.74	B:ARG 102[NH1]	CDRH3
8	A:ARG 419[NH2]	β19	3.59	B:ASP 74[OD1]	VH-FR3
9	A:ARG 419[NH1]	β19	2.89	B:ASP 74[OD1]	VH-FR3
10	A:ARG 419[NH2]	β19	2.53	B:ASP 74[OD2]	VH-FR3
11	A:ARG 419[NH1]	β19	3.28	B:ASP 74[OD2]	VH-FR3
12	A:ARG 469[NH1]	V5 Loop	3.56	C:ASP 90[OD1]	CDRL3
13	A:ARG 469[NH2]	V5 Loop	2.76	C:ASP 90[OD1]	CDRL3
14	A:ARG 469[NH1]	V5 Loop	3.10	C:ASP 90[OD2]	CDRL3
15	A:ARG 469[NH2]	V5 Loop	3.13	C:ASP 90[OD2]	CDRL3

Supplementary Table S4. Protein–protein interactions of the predicted FD22–B41 SOSIP trimer complex structure 2, analyzed by PDBePISA (ModelSeeds =1692646031, Ranking Score = 0.63).

Hydrogens bonds between B41 gp120 and FD22

##	B41-gp120	gp120 domain	Dist. [Å]	FD22	FD22 domain
1	LYS 178[O]	V2 Loop	2.16	TYR 75[OH]	VH-FR3
2	VAL 181[O]	V2 Loop	2.39	TYR 75[OH]	VH-FR3
3	PRO 183[O]	V2 Loop	3.20	GLY 26[N]	CDRH1
4	ARG 201[NH2]	V2 Loop	3.32	GLY 26[O]	CDRH1
5	ALA 281[O]	Loop D	2.84	VAL 107[N]	CDRH3
6	THR 283[N]	Loop D	3.45	GLY 105[O]	CDRH3
7	THR 283[OG1]	Loop D	2.69	GLY 105[O]	CDRH3
8	HIS 363[NE2]	CD4 BLP	3.20	TRP 56[O]	CDRH2
9	SER 364[O]	CD4 BLP	3.62	SER 53[OG]	CDRH2
10	SER 365[O]	CD4 BLP	3.34	SER 53[N]	CDRH2
11	SER 365[OG]	CD4 BLP	2.99	ARG 111[NH2]	CDRH3
12	SER 365[OG]	CD4 BLP	3.45	ARG 87[NH2]	CDRL3
13	GLY 366[O]	CD4 BLP	2.21	ARG 111[NH1]	CDRH3
14	GLY 367[N]	CD4 BLP	2.83	SER 31[O]	CDRH1
15	GLY 367[O]	CD4 BLP	2.72	SER 31[OG]	CDRH1
16	ASP 368[OD1]	CD4 BLP	2.55	TYR 32[OH]	CDRH1
17	ASP 368[OD1]	CD4 BLP	3.81	ARG 98[NE]	VH-FR3
18	ASP 368[OD1]	CD4 BLP	3.87	ARG 98[NH2]	VH-FR3
19	ARG 419[NH2]	C4	3.00	ASP 74[OD1]	VH-FR3
20	ARG 419[NH1]	C4	2.76	ASP 74[OD2]	VH-FR3
21	ARG 469[NH2]	V5	2.81	ASP 90[OD1]	CDRL3
22	ARG 469[NH2]	V5	2.81	ASP 90[OD2]	CDRL3
23	ASN 474[ND2]	C5	3.16	LEU 103[O]	CDRH3
24	ARG 476[NH1]	C5	3.34	LEU 103[O]	CDRH3

Salt bridges between B41 gp120 and FD22

##	B41-gp120	gp120 domain	Dist. [Å]	FD22	FD22 domain
**1**	LYS 178[NZ]	V2 Loop	3.05	ASP 74[OD2]	VH-FR3
**2**	ASP 368[OD1]	CD4 BLP	3.81	ARG 98[NE]	VH-FR3
**3**	ARG 419[NH1]	C4	3.29	ASP 74[OD1]	VH-FR3
**4**	ARG 419[NH2]	C4	3.00	ASP 74[OD1]	VH-FR3
**5**	ARG 419[NH1]	C4	2.76	ASP 74[OD2]	VH-FR3
**6**	ARG 419[NH2]	C4	3.35	ASP 74[OD2]	VH-FR3

Supplementary Table S5. Sequence alignment of the resistant autologous virus SGA14 Env gene with reference strain HXBc2.

```
HXBc2        MRVKEKYQHLWRWGWRWGTMLLGMLMICSATEKLWVTVYYGVPVWKEATTTLFCASDAKA  60
SGA14        MRVKGI-KKNCQRLWRWGTMLLGMLMICSATEKLWVTVYYGVPVWKEATTTLFCASDAKA  59
             ****   ::  :  **********************************************

HXBc2        YDTEVHNVWATHACVPTDPNPQEVVLVNVTENFNMWKNDMVEQMHEDIISLWDQSLKPCV  120
SGA14        YVTEVHNVWATHACVPTDPNPQEVVLENVTETFNMWENDMVEQMHEDIISLWDQSLKPCV  119
             * ************************ ****.****:************************
                                                                V2 Loop(178-198)
HXBc2        KLTPLCVSLKCTDLKNDTNTNSSSGRMIMEKGEIKNCSFNISTSIRGKVQKEYAFFYKLD  180
SGA14        KLTPLCVTLSCTDTDVRNTTNSSV-TINSTEAGMKNCSFNITTSINTKV-EDYALFYNLD  177
             *******:*.*** .  ..****    :    :. :*******:***. ** ::**:**:**
            V2 Loop (178-198)
HXBc2        IIPI--DNDTTSYKLTSCNTSVITQACPKVSFEPIPIHYCAPAGFAILKCNNKTFNGTGP  238
SGA14        VVQIKEKNDSTSYRLINCNTSVITQACPKVSFEPIPIHYCAPAGFAILKCNNKTFSGKGP  237
             :: *  .**:***:* .*************************************.*.**
                                                   LoopD (276-283)
HXBc2        CTNVSTVQCTHGIRPVVSTQLLLNGSLAEEEVVIRSVNFTDNAKTIIVQLNTSVEINCTR  298
SGA14        CTNVSTVQCTHGIRPVVSTQLLLNGSLAEEEVIIRSSNFSDNTKIIIVQLNKSVEIRCIR  297
             *********************************:*** **:**:* ******.****.* *

HXBc2        PNNNTRKRIRIQRGPGRAFVTIGK-IGNMRQAHCNISRAKWNNTLKQIASKLREQFGNNK  357
SGA14        PNNNTRKSVN--IGPGRAWYTTGQIIGDIRAAHCNLSASKWRDTLQNITEKLREQFKSKT  355
             *******  :.   *****: * *: **::* ****:* :**.:**::*:.****** .:.
                  CD4BLP (362-374)
HXBc2        TIIFKQSSGGDPEIVTHSFNCGGEFFYCNSTQLFNSTWFNSTWSTEGSNNTEGSDTITLP  417
SGA14        IIFKQPAPGGDPEITLHSFNCGGEFFYCNTSKLFNSTWYTNGNNTWNDSTEQENDTITLQ  415
              *: : : ******. *************::******:..  *    .:  : .*****
                                                      V5 Loop (458-469)
HXBc2        CRIKQIINMWQKVGKAMYAPPISGQIRCSSNITGLLLTRDGGNSN-NESEIFRPGGGDMR  476
SGA14        CRIKQIINLWQAVGKAMYAPPISGQIRCSSTITGLLLTRDGGETGDNTTETFRPEGGNMK  475
             ********:** ******************.***********::. * :* *** **:*:

HXBc2        DNWRSELYKYKVVKIEPLGVAPTKAKRRVVQREKRAVG-IGALFLGFLGAAGSTMGAASM  535
SGA14        DNWRSELYKYKVVKIEPLGVAPTKAKRRVVQREKRAVGTFGAMFLGFLGAAGSTMGAASI  535
             **************************************  :**:*****************:

HXBc2        TLTVQARQLLSGIVQQQNNLLRAIEAQQHLLQLTVWGIKQLQARILAVERYLKDQQLLGI  595
SGA14        TLTVQARQLLSGIVQQQRNLLRAIEAQQHLLQLTVWGIKQLQARVLAVERYLGDQQLLGI  595
             *****************.**************************:******* *******

HXBc2        WGCSGKLICTTAVPWNASWSNKSLEQIWNHTTWMEWDREINNYTSLIHSLIEESQNQQEK  655
SGA14        WGCSGKLICTTAVPWNASWSNKSLSDIWNNMTWMDWEREISNYTREIYTLIEQSQNQQEK  655
             ************************.:***: ***:*:***.***  *::***:*******

HXBc2        NEQELLELDKWASLWNWFNITNWLWYIKLFIMIVGGLVGLRIVFAVLSIVNRVRQGYSPL  715
SGA14        NELELLELDKWASLWNWFDITKWLWYIKIFIMIVGGLVGLRIVFAVLSIANRVRQGYSPL  715
             ** ***************:**:******:*********************.**********

HXBc2        SFQTHLPTPRGPDRPEGIEEEGGERDRDRSIRLVNGSLALIWDDLRSLCLFSYHRLRDLL  775
SGA14        SLQTHFPTQRGPGRPERIEEEGGERDRDRSETLANGFLILFWEDLRNLCPFSYHRLRDLL  775
             *:***:** ***.*** *************  *.** * *:*:***.** **********

HXBc2        LIVTRIVELLGRRGWEALKYWWNLLQYWSQELKNSAVSLLNATAIAVAEGTDRVIEVVQG  835
SGA14        LIVARVVELLGRRGWEVLRYWWNLLQYWIQELKNSAISLLNAATIAVAEGTDRVIEVAQR  835
             ***:*:**********.*:********* *******:*****::*************.*

HXBc2        ACRAIRHIPRRIRQGLERILL    856
SGA14        AYRAILNIPRRIRQGLERALL    856
             * ***  :************ **
```

Single-genome amplicons from the plasma of patient P27 were sequenced. The resistant autologous virus SGA14 Env sequences were aligned with reference sequences from HXBV2. V2 Loop, Loop D, CD4BLP, and V5 Loop are indicated.

Ab	Protein	*K*D (M)	*K*D Error	kon(1/Ms)	kon Error	kdis(1/s)	kdis Error
FD22	gp120$^{Bal.01}$	8.88E-09	2.48E-10	8.65E+04	1.58E+03	7.68E-04	1.62E-05
FD22	gp120$^{JRCSF.JB}$	1.81E-08	4.40E-10	4.98E+04	7.49E+02	8.99E-04	1.72E-05
FD22	gp120^{YU2}	1.21E-08	4.47E-10	3.46E+04	4.48E+02	4.20E-04	1.45E-05
VRC01	gp120$^{Bal.01}$	2.51E-08	9.66E-10	9.12E+04	3.10E+03	2.29E-03	4.12E-05
VRC01	gp120$^{JRCSF.JB}$	5.06E-08	6.69E-10	1.14E+04	6.84E+01	5.77E-04	6.79E-06
VRC01	gp120^{YU2}	9.47E-09	4.24E-10	1.79E+04	1.18E+02	1.69E-04	7.49E-06

Supplementary Fig. S1. BLI analysis of the binding affinity of FD22 for HIV-1 gp120$^{Bal.01}$, gp120$^{JRCSF.JB}$, and gp120^{YU2} using Octet RED96 instrument (Sartorius). The *K*D, kon and kdis values were generated by FortéBio Data Analysis 8.1 software.

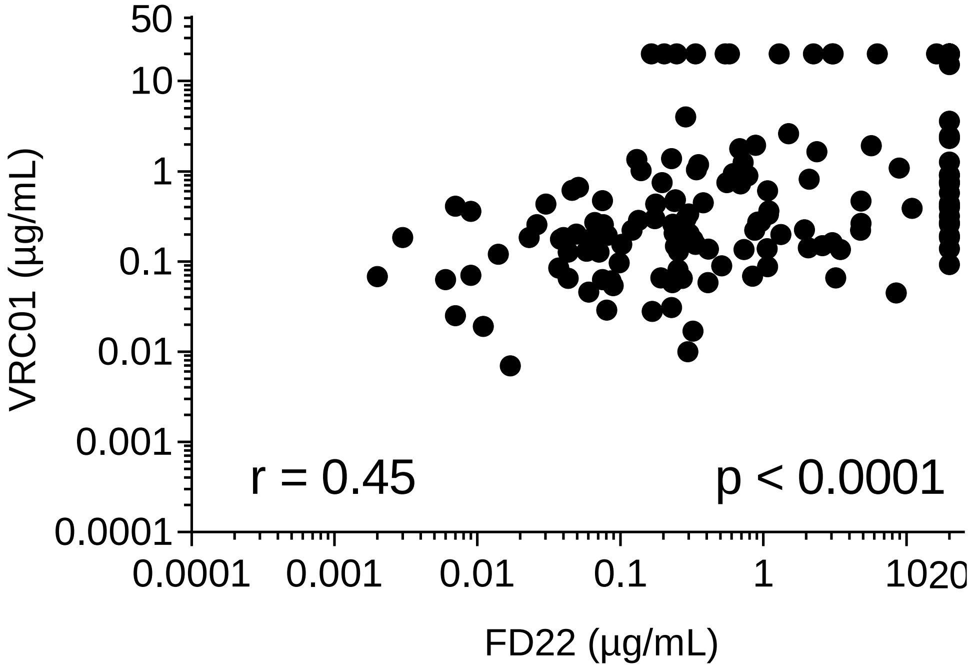

Supplementary Fig. S2. Correlation analysis of IC_{50} values between VRC01 and FD22.

A comparative analysis of neutralization potency was performed by correlating the IC_{50} values of FD22 and VRC01 across a panel of 145 HIV-1 pseudoviruses representing multiple clades. The IC_{50} values were plotted to assess the relationship between the neutralization profiles of these two antibodies. The correlation coefficient (R) and statistical significance (p value) were determined to evaluate the degree of similarity in their neutralization breadth and potency.

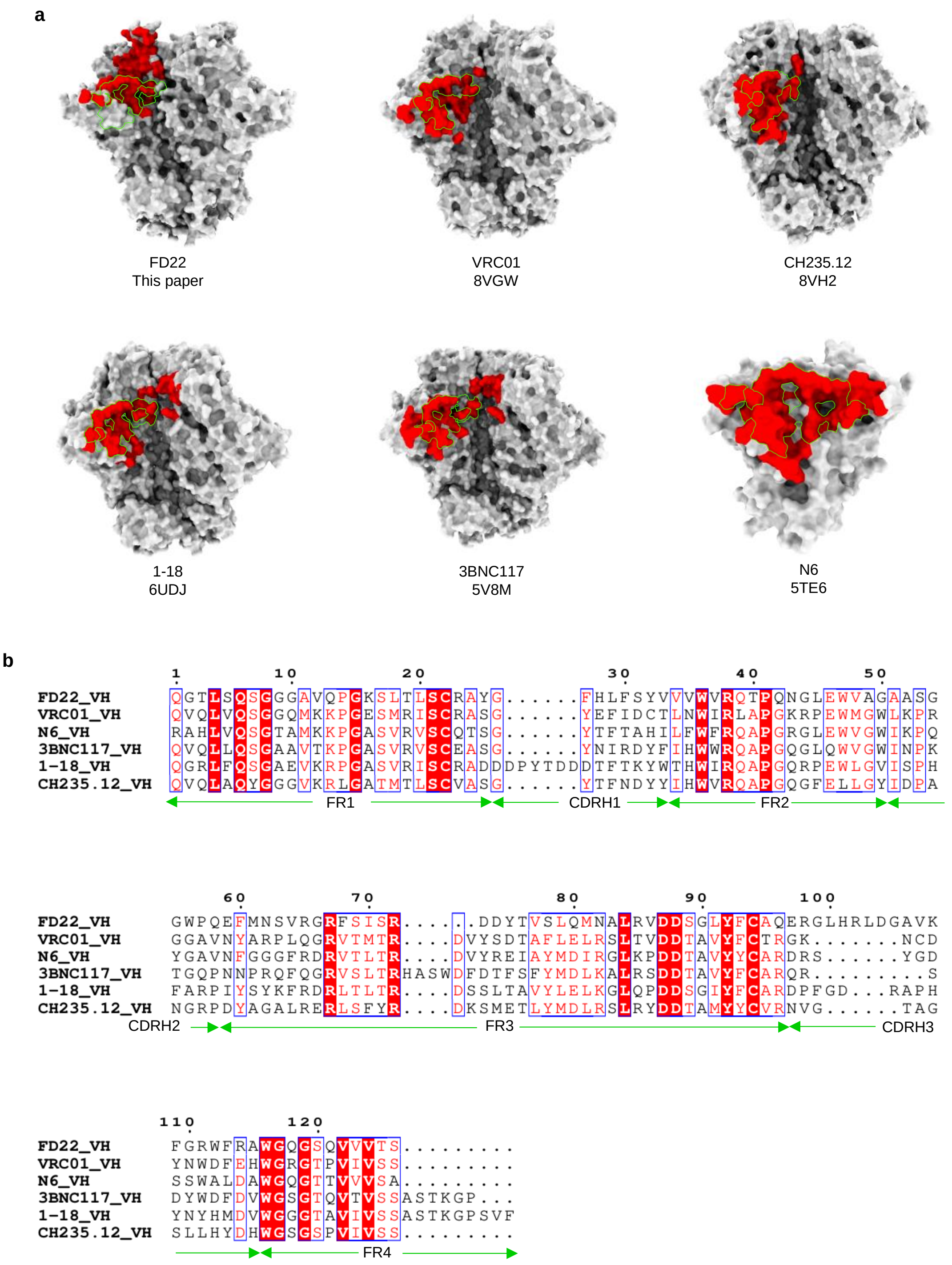

Supplementary Fig. S3. Comparison of epitopes of six CD4bs antibodies by structural prediction and sequence alignment

(a) Structural prediction of the binding of the CD4bs antibody to the HIV Env SOSIP trimer. The HIV Env SOSIP trimer is shown in surface mode, with epitope residues highlighted in red. The CD4bs is outlined with a green circle, indicating the region targeted by the six CD4bs antibodies.

(b) Sequence alignment of the heavy chains of CD4bs antibodies. Alignment of the

heavy chain sequences of six CD4bs-targeting antibodies, with conserved and variable regions and CDRs among the antibodies highlighted. This alignment helps to identify structural features critical for binding to the CD4 binding site on the HIV Env protein.

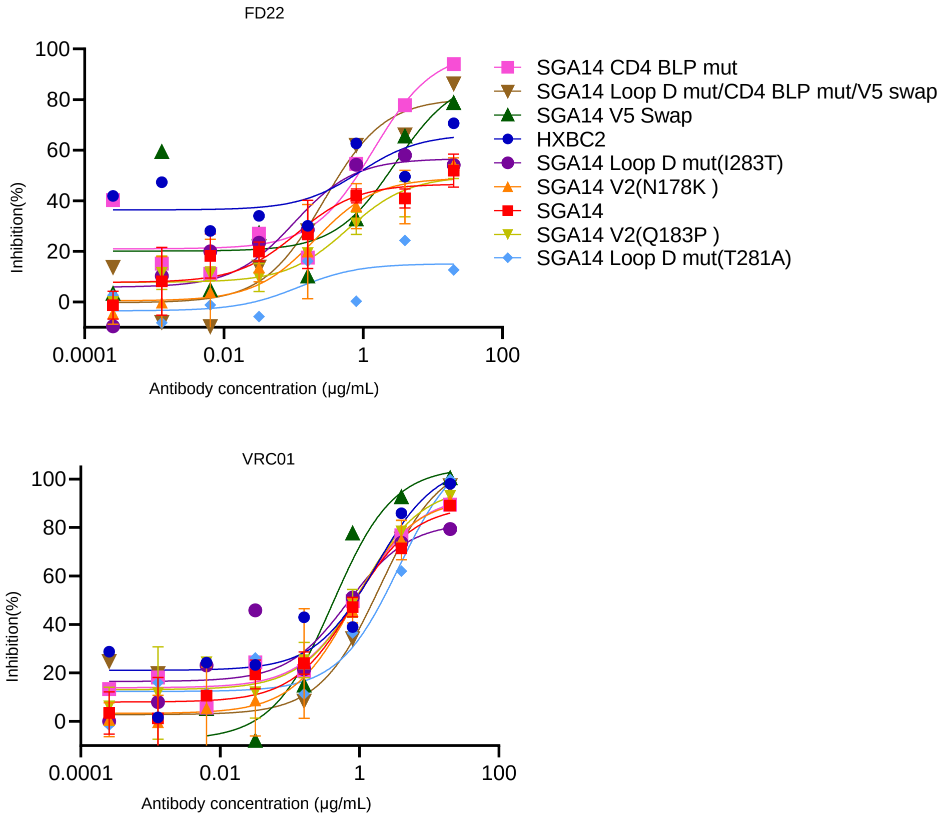

Supplementary Fig. S4. Neutralization curves of the resistant autologous virus SGA14 and its mutants with FD22. The resistant autologous virus SGA14, derived from P27, was engineered by substituting the V2 Loop, Loop D, CD4 BLP, and V5 Loop of HXBc2 Env into SGA14. VRC01 was used as a control.
